# Complete Genome Sequences of Two Isolates of Human Parvovirus 4 from Patients with Acute Encephalitis Syndrome

**DOI:** 10.1128/genomeA.01472-14

**Published:** 2015-01-29

**Authors:** Shantanu Prakash, Amita Jain, Akanksha Seth, Arvind K. Singh, Bhawana Jain

**Affiliations:** Virology Laboratory, Post Graduate Department of Microbiology, King George’s Medical University, Lucknow, Uttar Pradesh, India

## Abstract

Human parvovirus 4 (Parv4) is a relatively new virus. Association of this virus with any human disease is yet to be established. We detected human parvovirus 4 in the cerebrospinal fluid (CSF) of two patients presenting with acute encephalitis syndrome in northern India. This is the first report of the Parv4 genome sequence from northern India.

## GENOME ANNOUNCEMENT

Parvovirus 4 (Parv4) is a member of the Parvoviridae family, discovered in 2005 in the blood of a patient with symptoms resembling those of acute HIV infection (1). Parv4 is a small (20 to 22 nm in diameter) nonenveloped virus. The genome is single-stranded DNA of 5-kb length, which includes two open reading frames (ORFs). ORF-1 has 1,992 nucleotides (nt) encoding 664 amino acids (aa) and ORF-2 has 2,745 nt encoding 915 aa. ORF-1 forms a nonstructural protein (NS1) essential for viral replication, and ORF-2 divides into two structural proteins, VP-1 and VP-2 (2). PARV4 has been classified into 3 genotypes. Genotypes 1 and 2 are known to be present in North America, Europe, and Asia (3, 4), and genotype 3 is found in sub-Saharan Africa (5, 6). Until now, it has not been associated with any disease and its spread in the human population has not been clearly assessed, yet its presence in the cerebrospinal fluid (CSF) of two children from southern India (Bellary) presenting with encephalitis, arouse the possibility of its association in such cases (7).

Here, we report whole-genome sequences of two isolates of Parv4 viruses detected from the CSF of acute encephalitis syndrome (AES) cases (defined according to www.nvbdcp.gov.in/doc/revised%20guidelines%20on%20aes_je.pdf). CSF and serum samples of patients presenting with AES were collected and tested for the presence of possible etiological viruses (*viz.* Japanese encephalitis, dengue, herpes simplex 1 and 2, enterovirus, Varicella-Zoster, mumps, measles, Chikungunya, parvo B19, rubella, and West Nile viruses) prevalent in this area, in the Virology Laboratory, King George’s Medical University, Lucknow, India. In view of the report from Bellary (7), we picked up ten samples that tested negative for all the listed viruses and further subjected them to Parv4 detection by the real-time PCR method. Of these, two turned out to be parv4 positive and were planned for whole-genome sequencing by next generation sequencing (Ion Torrent). Viral DNA was extracted from the patient serum using the Purelink nucleic acid extraction kit (Invitrogen). A full-length genome sequence was obtained by conventional PCR using Phusion high-fidelity DNA polymerase. The library was prepared using an Ion Xpress Plus gDNA fragment library preparation kit according to the manufacturer’s protocol. Each library was sequenced on a separate Ion 314 chip (Life Technologies). Overlapping sequences were assembled manually and by an integrative genomics viewer (IGV) by mapping closely related genomes to establish the whole genome. The average coverage depth of the sequencing was more than 350×. Phylogeny reconstructions of the whole-genome sequences using the neighbor-joining method demonstrated that the human parv4 isolated from two North Indian patients clustered with genotype 2 reference strain and were 98% similar to the strains previously isolated in encephalitis patients from Bellary, south India (GenBank accession no. HQ593530) (7). The whole-genome sequences of newly discovered Parv4 will help researchers to get familiar with this virus.

### Nucleotide sequence accession numbers.

The sequence is available in GenBank under accession numbers KM390024 and KM390025.
